# ‘I felt the world crash down on me’: Women’s experiences being denied legal abortion in Colombia

**DOI:** 10.1186/s12978-017-0391-5

**Published:** 2017-10-23

**Authors:** Teresa DePiñeres, Sarah Raifman, Margoth Mora, Cristina Villarreal, Diana Greene Foster, Caitlin Gerdts

**Affiliations:** 1 Fundación Oriéntame/ESAR, Bogotá, DC Colombia; 2 Advancing New Standards in Reproductive Health (ANSIRH), University of California, San Francisco (UCSF), 1330 Broadway, Suite 1100, Oakland, CA USA; 3 Ibis Reproductive Health, 1330 Broadway, Suite 1100, Oakland, CA USA

**Keywords:** Abortion, Denial of abortion, Unsafe abortion, Colombia

## Abstract

**Background:**

In 2006, Colombia’s constitutional court overturned a complete ban on abortion, liberalizing the procedure. Despite a relatively liberal new law, women still struggle to access safe and legal abortion services. We aimed to understand why women are denied services in Colombia, and what factors determine if and how they ultimately terminate pregnancies.

**Methods:**

We recruited women denied abortion at a private facility in Bogota. Twenty-one participants completed an initial interview and eight completed a second longer interview. Two researchers documented themes and developed and applied a codebook to transcripts using ATLAS.ti.

**Results:**

Participants faced barriers, such as lack of knowledge of service availability and delayed pregnancy recognition, leading to denial. Five out of eight participants ultimately received abortions in public hospitals, due to support from partners and a robust referral system; nevertheless, they received poor care. Those who continued pregnancies endured stigmatizing events and inaccurate medical counselling at referral facilities. Several women contemplated illegal abortion though were afraid to attempt it.

**Conclusion:**

We propose the following recommendations: 1) increase awareness about availability and legality of abortion services to prevent delay and consequent denial; 2) provide counseling and referral upon denial; and 3) train providers in interpersonal quality abortion care.

## Plain English summary

In Colombia, abortion services are authorized by the federal government, with no specific gestational age limitations, except that services after 15 weeks must be performed at a high level facility. Despite legal availability, women in Colombia still face barriers to accessing safe abortion services. This paper seeks to understand why women are denied legal abortion services in Colombia, and what factors determine if and how they terminate a pregnancy after being denied services initially. We recruited 21 women immediately after they were denied services at a private facility in Bogota. These women reported delays in recognizing that they were pregnant and delays in determining where to go for legal abortion services. Those who were denied but ultimately terminated their pregnancy received support from partners and a robust referral system. Those who continued their pregnancies endured stigmatizing events and inaccurate medical counselling at referral facilities. Findings from this study indicate a need to increase awareness about abortion services to prevent delay and consequent denial, provide counselling and referral upon denial, and train providers in interpersonal quality abortion care.

## Background

In Latin America and the Caribbean, nearly 10 million unintended pregnancies were estimated to have occurred in 2012, 40% of which ended in abortion [1, 2]. A minimum of 10% of maternal deaths annually in the region are due to unsafe abortion, and about 760,000 women are treated for associated complications each year [3], including haemorrhage, sepsis, peritonitis, and trauma to the reproductive organs. In Colombia, one third of unsafe abortions result in complications that require medical attention, primarily heavy bleeding and incomplete abortion, and rates are even higher among women who self-induce using invasive techniques or seek help from an unqualified practitioner [4].

In 2006, Colombia’s Constitutional Court overturned a complete ban on abortion, decriminalizing the procedure in cases of rape or incest, foetal anomaly incompatible with life, and endangerment of the life or health of the woman [5]. The Colombian government released guidelines for abortion provision [6], adapted from the World Health Organization (WHO) [7], soon after the law was adopted but later annulled these guidelines due to challenges to the government’s authority to regulate abortion. More recent Ministry of Health technical documents now guide service providers on how to provide abortion services in the primary level, how to prevent unsafe abortion, and how to provide abortion counselling. The law does not include gestational age limits [8], but Ministry of Health protocol states that abortion services up to 15 weeks may be provided at the primary health service level, and services after 15 weeks must be performed at a higher level facility [6].

No concerted effort was undertaken to disseminate information about the change in legal status of abortion or to expand the number of providers, and by 2009, fewer than 3000 legal abortions had been reported, in contrast to an estimated 320,000 to 450,000 illegal abortions annually [9–11]. Approximately half of abortions in Colombia are induced using misoprostol, a low-cost medication used to induce an abortion or miscarriage, and the other half by non-misoprostol methods estimated to be evenly provided by medical doctors, other health professionals, or traditional providers [4]. Medical providers rely more on dilation and curettage (D&C) than on manual vacuum aspiration (MVA) [10], despite WHO recommendations to use MVA in the first trimester [12]**.** While there is little available data on abortion complications, an estimated 93,300 Colombian women sought post-abortion care in 2008 (1/3–1/4 of total procedures) [10]. Such a high proportion of total procedures requiring post-abortion care may indicate that a large proportion of abortions happen outside of the formal health system and result in women seeking additional treatment [4]; post-abortion care rates may be higher than necessary because many women may not be well informed about the normal process of an abortion using misoprostol nor what sequelae of a medication abortion require medical attention [13, 14].

Even where abortion is legal, poverty, stigma, and distance from a provider prevent women from accessing safe abortion services, among other factors [2, 15]. Despite a relatively liberal law in Colombia, which permits abortion free of charge in the public sector without a gestational age limit, barriers to accessing quality abortion care remain, especially later in pregnancy [16]. Previously documented barriers include: lack of referral protocols, narrow interpretation of the health exception (excluding mental health), stigma, lack of awareness about legal services, financial barriers, and delays to care [16, 17]. These may lead to the denial of services for women, particularly at primary health facilities which have gestational age limitations. As is the case in other contexts where barriers to legal abortion care exists, some women may seek services elsewhere, either at another facility, through self-induction, or with the help of an informal sector provider [18–20]. Recent Global Turnaway Studies in Nepal [21], South Africa [22], and Tunisia [23] have shown this to be true in other settings. However, little research has been done in Colombia to understand why women are denied legal services, whether they seek services following denial, and what factors enable them to obtain services after denial.

We aim to answer the primary question: among those denied abortion care, what delays and barriers did they face? We additionally explored the factors that enabled or prevented women from seeking safe and legal services after being denied care and whether women used or considered using informal sector abortion methods outside the formal health system after denial. This study was conducted as part of the Global Turnaway Studies; other participating countries include the United States [24, 25], Bangladesh [26], South Africa [22], Nepal [21], and Tunisia [23].

## Methods

In September 2013, women denied abortion due to gestational age limits at Fundación Oriéntame, a private not-for-profit clinic in Bogotá, were recruited for in-depth interviews. Oriéntame is the largest provider of abortions in the country and partners with an on-site legal advocacy group to provide information to women about legal abortion. While there is no gestational age limit in Colombia, at this time Oriéntame was unable to provide abortion past 15 weeks gestation, because they were not a secondary care facility [27]. A previous study demonstrated that 2% of women surveyed at the clinic did not receive the abortions they sought, due to advanced gestational age [19]. Trained interviewers—two nursing assistants and one psychologist—approached women after their medical visits, explained the study, obtained informed consent and screened for eligibility. Eligibility criteria included denial of abortion due to advanced gestational age and ability to speak Spanish. If eligible, interviewers conducted initial interviews, about 15 min in duration, at the time of recruitment in person at the clinic. Interviewers contacted women 2 months later for a second longer interview, about 30–45 min in duration, conducted by telephone. Interviews were conducted by telephone due to resource and time constraints. The two-month time frame allowed researchers to learn about women’s experiences after denial; it was necessary to provide participants time to decide on their next course of action. Participants were compensated with a grocery store certificate worth 36,000 Colombian pesos (~$20 in 2014).

The initial interview guide included open-ended questions about clinic visit, including reasons for denial, reasons for seeking abortion, and factors that contributed to delay seeking services. The longer interview guide included a review of the initial abortion seeking process and questions about the respondent’s reactions to denial, actions taken following denial of care, experiences with referral and subsequent counselling, knowledge of legal and illegal abortion methods, pregnancy outcomes, and overall quality of abortion care. In this context, quality of abortion care was assessed through the perceptions of the women, including satisfaction with the services received, interpersonal care provided, presence of complications or pain related to the procedure, and whether participants recommend the service to others.

Interviews were conducted in Spanish, recorded, transcribed, and translated to English for analysis. Data were analysed using a qualitative content analysis approach, using a consistent set of codes to organize text with similar content after data collection was completed, transcribed and translated. A priori themes were identified, based on code types and results from previous studies about abortion denial and barriers to abortion in Colombia [16, 21–23]. Additional codes and sub-codes were generated iteratively according to emergent themes throughout the coding process. One coder conducted analysis in Spanish, generating initial codes and documenting emerging themes. A second coder analysed data in English, generated codes and validated themes against those created by the first coder. Researchers analysed all qualitative data using Dedoose 5.0.11 (SocioCultural Research Consultants: Los Angeles, CA) and synthesised socio-demographic data using Excel. Coding and transcripts were analysed repeatedly as necessary, and referred to throughout the analysis and writing process. The entire team reviewed key themes and illustrative quotations throughout the process. A study identification number and pregnancy outcome, when available, are included in parentheses following each quotation in this manuscript. Facility names have been retracted for confidentiality. The Ethics Committee at Oriéntame and the Committee on Human Research at the University of California, San Francisco approved this study.

## Results

### Summary

Twenty-three women were recruited and 21 were eligible for participation in the study. Most participants were 19–24 years old; three were 16–17 years old. Most women (15) lived with their parents; three lived independently, and three lived with their partners. Twelve out of 21 participants had been pregnant before and 11 had at least one child. Almost all were 15–20 weeks gestational age at denial, with one woman at 30 weeks**.**

Second interviews were conducted 2 months after recruitment with eight of the 21 participants who completed initial interviews (ID1, ID2, ID3, ID4, ID22, ID27, ID28, and ID29). The remaining 13 participants did not respond, had a non-functioning phone number, were no longer in Bogotá, or did not want to participate in a second interview for unknown reasons. Some women declined phone interviews because it was difficult to find a quiet and private space to talk.

Below we present results from initial interviews regarding delays and barriers to seeking legal abortion services (part 1). Subsequently, we present factors that enabled or inhibited participants in seeking legal abortion care after denial and their knowledge of and experiences with self-induction and illegal methods (part 2).

### Part 1: Delays and barriers to seeking and accessing abortion services

Participants reported delayed recognition of pregnancy, lack of knowledge about legal abortion availability, logistical barriers, and/or need for time to decide. Six of the 21 participants said they did not realize they were pregnant until the second trimester, due to lack of pregnancy symptoms or irregular menstruation. One participant, who was 18 weeks upon denial, said, ‘…I haven’t had my period for about 5 months. I thought the injections I was using for contraception had made my menstruation irregular. That made me think everything was normal’ (ID30). Another, who sought abortion at 16 weeks, said: ‘My period came normally, but I realized that I was pregnant when I started to see I was looking fat and that my belly was hard. I took a pregnancy test but it was negative…later I did a blood test and it was positive’ (ID13).

Some participants did not know about the abortion law: ‘I thought [abortion] was illegal, that it was denying life to a human being and no one could do it legally’ (ID29). None were aware about the health exception, which includes mental health: ‘I hadn’t even thought of the possibility that if you were in a bad emotional state, like I was, you could find legal support for the procedure. I didn’t know that. …They don’t provide information about it, because of the Church and people’s ideas, so many taboos’ (ID1).

Logistical complications also delayed abortion seeking, including care-taking responsibilities, work, or lack of resources. One participant recalls: ‘I confirmed [that I was pregnant] a month before. I didn’t come in earlier because I didn’t have money. …When I came they told me I was 11 weeks pregnant. I made an appointment for August 30 but I didn’t come because I didn’t have all the money. …by the time I came in…they told me I was at 16 weeks’ (ID27). Many did not know where to seek services: ‘There is a lack of information, lack of awareness, of support, of counselling. Some people may know about the clinic, but lots of people don’t. So, with more information, more advertising, more use of media, people will know what to do in this case and not wait so long’ (ID29).

Some participants delayed because they needed time to make a decision about the pregnancy. One explained: ‘…that’s why I took so long. I told him that we should think about it. I searched for shelters for mothers in my situation. I thought about all these things, about school, and what I could give the baby’ (ID28). Another participant needed an abortion for health reasons but still took time to make the decision: ‘Of course, the decision was not easy. I got to the last week, I mean I waited a week more… After that week no hospital in Bogota would have done it. Since I was young, I was afraid of abortions’ (ID22).

Lastly, several participants felt devastated when they were denied abortion services. One said immediately after: ‘I am panicking…I can’t see myself as a mom. I hope something can be done’ (ID2). Another said: ‘When they said they couldn’t perform the abortion, I felt the world crash down on me’ (ID27). An 18-year-old, who ultimately continued her pregnancy due to pressure from her partner and her mother, said: ‘I am very sad because all of my plans have changed. I wanted to study next semester and now I have to wait six months. It’s for these reasons that I didn’t want a baby right now. It’s difficult. I will no longer be able to be young’ (ID4). Finally, a participant, whose husband left her when he learned about the pregnancy, said: ‘It destabilizes many things. …I won’t be able to study; that life plan will have to wait until the baby is older’ (ID1).

All 21 respondents were referred to an advocacy group based in-house at the clinic, which provided legal advice for seeking abortion in the public sector. Participants were advised to present their request for abortion within the context of one or more of the circumstances sanctioned by Colombia’s abortion law.

### Part 2: Factors that enable or inhibit access to safe and legal abortion care

#### Partner involvement in decision-making

Four out of five participants who successfully terminated were no longer in a relationship with the man involved in the pregnancy when they pursued abortion. As a result, the men were either not included in the decision-making process or did not oppose abortion. One participant said: ‘No, it was a passing thing. We only went out for a month. He left. …So he never found out’ (ID29). Another explained: ‘My partner knows and doesn’t want to have it. We don’t have a relationship any more. We broke up and I am not going to see him again’ (ID22). The participant who terminated her pregnancy while still together with her partner explained that her partner also wanted her to have an abortion: ‘He really didn’t want to have it… He said, “No. We’re not prepared to have children now. We’re in college, we’re just starting out.”’ (ID28).

The three participants who carried to term said they lacked support from their partners in seeking abortion. One explained: ‘I asked him and he told me he didn’t agree because it’s not the baby’s fault’ (ID4). Another said: ‘I had a serious argument with my partner. He told me it was my fault for spreading my legs… (ID3). One participant’s relationship with her partner deteriorated after she told him about the pregnancy: ‘When I told him I was pregnant, I never thought of having an abortion. …I always assumed that he was going to support me; but no. It was the moment for him to tell me, “I am seeing someone else and I don’t want the responsibility of more babies. You are taking away my chances to study, to travel, by bringing so many babies into the world.” These things made me sad, anguished’ (ID1).

#### Legal support and counselling

All eight participants who completed a second interview confirmed they received legal support from the advocacy group where they were referred upon denial. Five of the eight ultimately obtained abortions at public hospitals. The remaining three participants continued their pregnancies.

Those who obtained abortions explained that the legal support and counselling was crucial to their success. After being counselled, one participant said she was able to effectively advocate for herself and navigate the complex system:

*I spoke with the lawyer, who explained to me the reasons for which one could have an abortion and told me to go to the [hospital]. I went there, talked to the receptionist and said it was an emergency... [the doctor] asked how many weeks I was at, and I told him that I was at 19 weeks and that I wanted an abortion. He asked me if my reason was within one of the three legal indications, and since the law contemplated psychological as well as physical health, I needed to be evaluated by a psychologist to see if he could do the procedure. The psychologist evaluated me and she was the one who approved that my mental health was at risk.* (ID28)

Another participant described how legal counselling empowered her to make a well-informed decision:

*[The psychologist] encouraged me to talk about it calmly, to not feel bad, that it was a decision I had made and that no one was going to judge me because it was my body and I was the person who was going to give that child everything he deserved and no one else was going to help me…I should feel calm and open up to her. I felt supported. If I was sure, who were they to judge me?*

*[With legal counselling] you’re sure and you have support to back you up. You have more people helping you and you do not doubt yourself. What should I do? Where should I go? On the contrary, you have counselling, solid support, you can say what you want or don’t want. You know the risks.* (ID29)

#### Stigmatizing experiences at referral facilities

Three out of eight participants who completed a second interview did not ultimately obtain abortions, despite legal counselling and support (ID1, ID3, ID4). Specific encounters with providers and another patient at the referral facility influenced them to ultimately decide against abortion. ID1 was confident in her initial decision (her husband was leaving her, she had a two-year-old child and she was overwhelmed by the idea of raising two babies alone), but she changed her mind after the doctor questioned her. She recalls:

*When I arrived, I thought I was sure. But when the whole process started, no. Something that happened was that I told the doctor I could feel fast heartbeats in my stomach. He felt my stomach and said the baby had tachycardia. He began to tell me about how babies can sense when they are in danger, things like that…. He told me that it was very possible that they would not be able to do the procedure because of how far along I was… He told me that he didn’t recommend it but that he was going to refer me to another place where they dealt with these cases.* (ID1)

ID3 was also determined at first, but later became ‘destabilized’ after her ultrasound:

*Knowing that it’s not going to be a happy baby or that it’s not going to have a good future…I think that the best decision in that moment is to end the pregnancy. … Just imagine, after you see an ultrasound where the baby is totally formed, where you hear his heart, where you know that it’s a little person that only you can feel. Obviously, that destabilizes you emotionally in an inexplicable way. No one can understand that, except the person who is in that situation. Despite all that, I tried to say no.* (ID3)

ID3 met with a lawyer and sought an abortion at a hospital, but finally decided against it. She explained:

*I saw some girl that was there for the same reason as me. She was worse off than me, because my parents supported me, despite the fights.... My parents knew about the pregnancy since I was six weeks and they never turned their back on me, never. … My decision now to continue with the pregnancy is due to the support I've had from my parents. When you hear someone who really [has no support]... you say, oh yeah, I will be okay if that person is at my side.... Something had to happen that day to make me react. I left and told my partner …It’s the best decision I’ve made in my life, even though I know that abortion should be legal in this country. I support it. I’ve had an abortion before. I have been through these things, which is why I support abortion.’* (ID3)

#### Poor interpersonal care, despite access

None of the five participants who ultimately obtained abortion suffered from medical complications; however, most experienced poor treatment and felt stigmatized. One participant explained: ‘For me it was super difficult. To begin with, I’d never been to a [health facility] alone. ..It was a shock. On the way ...there are people who pass out fliers that say “unwanted pregnancies.” Everything goes through your mind. There are ladies giving away religious icons and anti-abortion propaganda… it’s an emotional shock’ (ID2). Inside the hospital, she endured poor treatment from providers:

*…It was really hard to hear children crying nearby in the birthing rooms, to hear mothers pushing. …At around 11:00 at night, I started having strong contractions. The nurse who received me that night came in and performed a really rough examination. …Obviously they didn’t approve of what I was doing and they wanted to get back at me. I was really in pain; I was screaming. They did another psychical examination and that was when my water broke and then I felt the foetus being expelled...*

*About two hours earlier a woman came in… She was two months pregnant and it was a high-risk pregnancy. She expelled it. It was super tiny and everything happened right next to me. The woman started crying because she wanted to have her baby. When the nurse picked up the little foetus to take it to pathology, the woman was crying. The nurse glanced at me and said, “Ironic, don’t you see? She wants a baby and you’re tossing one out.”*

*I was really hurting. The pain made me think about other things. At the moment of the expulsion, the nurses picked up the foetus. They told me it was alive and the woman [next to me] started crying. I did too. I didn’t say anything to the nurse. I was feeling really bad. …The woman looked at me and cried and I felt this emotional weight. I cried, “What can I do.” I was in the bed bleeding.* (ID2)

Some participants navigated significant bureaucratic challenges at the hospital, including inefficient referrals and unnecessary paperwork. One 19-year-old woman describes:

*I had to write a letter requesting authorization … explaining my reasons, and stating that I was totally sure, with photocopies of my documents. … They called on Thursday and told me to go on Friday to the office to pick up the authorization form …. I went but the form said, “Appointment for gynaecology and obstetrics.” I took the form to gynaecology where they told me it was only an authorization to schedule an appointment. I went to schedule an appointment and they gave me one for ten days later. …I went at 7:30 in the morning [the next day] and explained my situation. I showed the authorization form. The department head was there and I told her everything. Super rude. She said, “But that’s not the way it’s done. Show me the piece of paper that says you have one of the legal causes.” Everything had been sent … I already had the authorization but they wouldn’t receive me.* (ID2)

Another participant was hospitalized for 2 days without receiving care, during which time her providers disrespected her and criticized her decision to seek an abortion:

*I was there but they didn’t do anything. They just sent me to the psychiatrist and told me it was a crime… They really treated me bad. The whole hospital found out—everyone. …they were all talking about it. All the nurses walked by and looked at me. …They asked me why I wanted to do it, if I didn’t care. I mean, I didn’t have to explain…it’s my decision and what business is it of theirs? They’re strangers; I don’t know them. All the doctors of all the shifts found out that I was there for two days. They even called the police and everything because they said it was illegal and that I had to make a statement to the police. … It was intense because I was feeling bad, with all the people there judging you without knowing your condition. I felt bad.* (ID27)

This participant went to a different hospital, where a provider told her he couldn’t help her “because of his personal integrity.” She returned the next day and obtained an abortion from a different provider, but reported how difficult it had been: ‘…they held [the foetus] in their hands and everything. I saw it and I felt bad. It really hurt. I started crying and after leaving the hospital I couldn’t sleep’ (ID27).

One participant, who was placed in a maternity hospital room, said that when the doctors realized she was there for an abortion rather than delivery, they treated her differently:

*…they gave me a bed with the other moms, like a normal patient. But then came the shock of seeing all of them with their babies and me, with an abortion. Then they started to treat me poorly. They refused to give me [pain] medication. They delayed everything… I was very sore physically and emotionally and I couldn’t make them be more considerate of my situation.* (ID22)

As a result, she recommended provider training to prevent poor treatment for other women seeking abortion: ‘I think they need to hire, or make [providers] more aware and sensitive, or carry out a medical education campaign… starting in med school. …Because they swear to protect life even if people don’t want to live and they make people be born even if they don’t want to’ (ID22).

Despite the poor interpersonal care in public hospital settings, none of the participants expressed feelings of regret about their decision. One said: ‘It hurts, but at least now I can sleep, I can be peaceful. It wasn’t easy at all, but I don’t regret it either’ (ID27). Another said: ‘When I finally managed to have the abortion, I was calm. And now I think that if I hadn’t had an abortion, I would be really bad off… because I am still a young woman and sex is a physiological, mental, and sentimental need. …I am grateful for the women’s movements that have fought for rights and to open our thinking. Unfortunately, there is guilt that you can’t erase; it stays’ (ID22).

#### Self-induction and illegal abortion

Many women said they considered self-inflicting pain or injury, self-inducing abortion, or visiting an illegal provider before they came to Oriéntame. One participant said: ‘[I thought of] poisoning myself, something, damaging my stomach somehow to see if it worked’ (ID29). Another said: ‘I don’t know if I was to commit suicide because I am very afraid of death. But I felt desperate and thought, “If I cut my veins, maybe I can damage the baby.” That way, it might not be an abortion. I would just lose it. I didn’t eat to see if I would lose it…’ (ID28).

Most participants said they heard about pills for self-induction and a couple attempted to obtain them. One participant said: ‘You rely on information from your friends and it’s a chain. Everyone goes to school or anywhere with rumours and they tell you… I was 17. I thought, “My parents don’t support me. My partner is very young…” At that moment, you don’t think about anything…I had information. I had access [to a friend’s pharmacy]’ (ID3). Another participant explained: ‘We researched and, because of the gestational time, we found some pills online… They’re super easy to buy. Each pill costs 15 thousand pesos. But they asked me, “How far along are you?” I said “two long months.” And they said they couldn’t sell them to me…’ (ID2). According to one participant, self-induction was a last resort: ‘There are many women who are unaware so we resort to other things. It is difficult because you don’t have an open space to discuss sexuality and get counselled about these topics. They only talk about how to protect from diseases and how to use contraception… I thought that if nothing could be done, I would take the risk because I really didn’t want to be a mother’ (ID2).

Most participants were afraid that alternative methods would not work or would be harmful. One participant recalled: ‘Of course. I thought about the possibility of Cytotec. I checked out clandestine places on the web. [City] is full of those places. I went to one, went in, and said to myself, “I could die in here.” Nothing was good in that place’ (ID3). Another said: ‘…I started researching a lot of things, online, talking to friends, without telling them I was pregnant. I just listened and learned…about the Cytotec pills they can buy. They spent a lot of money on those pills and they didn’t work because the baby was still there.’ (ID1). A third participant said: ‘I heard about [pills] in grade school and in college too. But I couldn’t. I heard that when you do that, you had to be with someone else in case something bad happens and be close to a hospital if anything happens. …I decided not to because I don’t want to die’ (ID28). One of two participants who were approached by illegal providers outside of a clinic explained: ‘He was pulling me and I got scared. I told him, “I’m going to call the police. I am just here for an ultrasound.” And he said, “You’re lying. You’re going to have an abortion. There’s a place where they charge half as much for the same things, with a doctor”’ (ID1).

## Discussion

We aimed to explore the barriers women face in accessing abortion care, the factors that enable or prevent women from seeking safe and legal services after denial, and the prevalence of informal sector abortion attempts after denial.

Results confirm prior research that preventable barriers to care, such as lack of knowledge of services, logistical barriers, or delayed pregnancy recognition, delay women from seeking abortion services earlier in their pregnancies. These delays, which can carry women past 15 weeks gestation, the gestational age limit for the study clinic, lead to unnecessary denial of services and, further, make it more difficult for some women to obtain wanted abortions, particularly in cases where they must defend their decision to partners and providers at later gestation.

Our findings suggest that key factors influencing whether or not women obtain a wanted abortion following denial include: partner support, legal counselling and referral at the moment of denial, medically accurate counselling at all points-of-care, and quality interpersonal care from providers. Women who chose not to discuss the pregnancy with their partners had a more straightforward path to care than did women who had to manage partners’ resistance; women whose partners were supportive of abortion were more likely to obtain care. Legal counselling from the on-site advocacy group played an essential role in enabling participants to effectively navigate a complex and bureaucratic health system, understand the law and its implications, and ultimately arrive at the next point of care prepared to advocate for themselves. Other studies show that women who are denied care without explanation or referral may be left with no option but to carry the unwanted pregnancy to term [21–23, 26].

Partner support and robust referral programs are not necessarily sufficient to ensure access to abortion. Stigmatizing experiences at referral facilities and poor interpersonal treatment from some providers ultimately prevented some participants in this study from obtaining wanted abortions. In at least two of the three cases where participants decided to carry to term, providers manipulated patients by exposing them to the foetal heartbeat and ultrasound images, and by advising them to continue the pregnancy based on medically inaccurate information. Furthermore, those who obtained abortions following denial endured physical and psychological abuse from providers and hospital staff, possibly due to inadequate training about the law and social stigma associated with abortion. Some clinicians may be required to perform abortions despite lack of training or personal objection. Comprehensive provider training should not only cover technical skills but also interpersonal quality care techniques, which treat all women, including those who have unwanted pregnancies, with respect and empathy [2]. The WHO considers interpersonal interactions to be part of quality of care, as evidenced by their definition, which includes the following key dimensions: effectiveness, efficiency, accessibility, acceptability/patient-centeredness, equity, and safety [28].

Many women were aware of self-induction, including with misoprostol, and some were aware of informal sector providers. This is unsurprising given estimates that over 99% of abortions in Colombia are performed outside of the formal health system and over one-half of these are performed using misoprostol [18].

It is important to acknowledge the limitations of our analysis. First, because study participants were sampled from a formal-sector abortion facility, it is highly likely that their knowledge of and experience with informal sector abortion is under-representative of that of all women in Colombia, particularly rural and poor women who bypass the formal sector altogether. In addition, as anticipated with an exploratory qualitative study, these findings are not generalizable or necessarily representative of all women in Colombia. Our results do not include the experiences of young women under 18 years or of women who seek abortion outside facility-based care.

## Conclusion

To our knowledge, based on a review of the literature and consultation with local experts, this is the first study to examine the experiences of women denied legal abortion in Colombia. Our findings highlight the need for: 1) public awareness campaigns about the availability and legality of abortion services in Colombia to prevent delay and consequent denial; 2) provider support and referral to patients if and when they are denied services for any reason; and 3) training on compassionate care for all providers and medical staff who encounter abortion-seeking patients. These improvements will help to ensure that women are able to obtain timely, safe, effective, and non-judgmental abortion care when needed. Similar research is needed to better understand the experiences of women denied abortion services across the country, particularly given that Oriéntame is likely the best case scenario for abortion care in Colombia. In the long term, systematic quantitative data collection would enable research on the health and socioeconomic consequences of legal abortion, illegal abortion and childbirth in Colombia.

## Abbreviations

D&C: Dilation and Curettage
MVA: Manual Vacuum Aspiration
WHO: World Health Organization
